# Thermal degradation and flame spread characteristics of epoxy polymer composites incorporating mycelium

**DOI:** 10.1038/s41598-023-45097-0

**Published:** 2023-10-19

**Authors:** Nattanan Chulikavit, Tien Huynh, Akbar Khatibi, Raj Das, Everson Kandare

**Affiliations:** 1 School of Engineering, RMIT University, Bundoora, VIC 3083 Australia; 2 School of Science, RMIT University, Bundoora, VIC 3083 Australia

**Keywords:** Biomaterials, Biomaterials

## Abstract

Although bioderived flame retardants are environmentally sustainable and less toxic, their impact on the thermal stability and flammability of polymers remains poorly understood. In this study, we assessed the influence of mycelium on the thermal stability and flame spread characteristics of epoxy through thermogravimetric analysis, Fourier transform infrared spectroscopy, the UL94 flammability test, and scanning electron microscopy. We observed a decrease in the maximum mass loss rate temperature when mycelium was incorporated into epoxy, indicating an earlier onset of thermal degradation. The inclusion of mycelium increased char yields above 418 °C due to mycelium’s inherent char-forming ability. However, mycelium did not alter the thermal degradation pathway of epoxy. Furthermore, according to the UL94 test results, the incorporation of mycelium reduced the flame spread rate compared to that of neat epoxy. These findings contribute to our understanding of the interaction between bioderived flame retardants and polymers paving the way for the development of more sustainable fireproofing materials.

## Introduction

When polymer matrix composites are exposed to high temperatures during a fire, they ignite, thermally decompose and burn. The thermal decomposition of polymer matrix composites releases toxic volatile compounds that can impair visibility, and cause death by asphyxiation. For example, the thermal decomposition of epoxy produces hazardous volatiles such as formaldehyde, phenols, volatile organic compounds (VOCs), carbon monoxide (CO), carbon dioxide (CO_2_), and carbon soot (smoke)^1–3^. Formaldehyde is a toxic gas that irritates the respiratory system and can also cause various other health effects^4,5^. Phenols also irritate the respiratory system and have toxic effects on human and animal health^6^. VOCs, which may include hydrocarbons, can cause dizziness and they are potentially carcinogenic^7,8^. Incomplete combustion of epoxy can produce highly toxic CO, leading to asphyxiation when in elevated concentrations. Additionally, the thermal decomposition of epoxy can release acrid smoke and irritating fumes from the thermal decomposition of a thermal degradation by-product, 4,4ʹ-methylenediphenol^9,10^. Heavy smoke and irritating fumes hinder evacuation in fire situations causing death or serious injury.

To improve fire resistance and reduce the volume of toxic gases and smoke released during the thermal decomposition of polymer composites, flame retardants are typically added to the polymer matrix^11^ or thermal protective surface coatings^12,13^. Various classes of flame retardants can be integrated into polymers, each of which has its own mechanism of action, and advantages and disadvantages. In the past, halogenated flame retardants have been widely used for their effectiveness in interrupting combustion within the gas phase^14^. Halogenated flame retardants include compounds containing bromine or chlorine, such as polybrominated diphenyl ethers (PBDEs) and chlorinated paraffin. When subjected to high temperatures, halogenated flame retardants release free radicals that interrupt the flaming process by combining with by-product radicals which would otherwise propagate the fire. However, despite their effectiveness in reducing the flame spread and smoke generation, halogenated flame retardants have been phased out due to their potential environmental persistence and toxicity, as well as their contribution to ozone depletion^15–17^.

Halogenated flame retardants have now been replaced by phosphorus- and nitrogen-based compounds such as phosphates, phosphonates, phosphazenes, melamine, melamine polyphosphate, and melamine cyanurate^18,19^ as well as inorganic salts such as aluminium hydroxide, magnesium hydroxide, layered double hydroxide, and hydroxy double salts^20–22^. Phosphorous-based flame retardants act through a combination of mechanisms, including char formation, dilution of volatile components, and flame quenching. While the environmental persistence and toxicity of phosphorous flame retardants may be substantially lower compared to halogenated compounds, higher loading levels are required which may adversely impact the mechanical properties of the host polymer matrix^23,24^. Nitrogen-based flame retardants release inert gases during decomposition, diluting oxygen, and combustible volatiles, and promoting the formation of a protective char. While effective in reducing smoke and toxic gas emissions, these compounds have limitations in achieving high flame retardancy levels and they are also sensitive to moisture. Inorganic flame retardants release water vapour when heated, thereby having a cooling effect while also diluting combustible gases^25–27^. These compounds contribute to char formation through the formation of metal oxides within the condensed phase. Inorganic flame retardants are less toxic and highly thermally stable, but higher loading levels may be required, impacting the mechanical properties and processability of modified polymers.

Recently, the focus has shifted to a new class of flame retardants derived from renewable biomass sources, such as plants or agricultural waste. Bioderived materials with potential flame retardation effects such as mycelium are more sustainable and environmentally friendly, with versatile multifunctional properties^28,29^, compared to fossil fuel-derived or synthetic inorganic flame retardants. Biodegradable flame retardants can be broken down by natural processes, reducing their long-term environmental impact. Moreover, the by-products generated from the thermal decomposition of bioderived materials, such as water and CO_2_ have reduced toxicity. Despite holding promise as more sustainable alternatives to traditional flame retardants, further research is required to improve our understanding of the flame retardation mechanisms inherent in bioderived materials.

In this paper, we employed thermogravimetric analysis (TGA), Fourier Transform infrared (FTIR) spectrometric analysis, and scanning electron microscopy (SEM) techniques to explore the interaction with and influence of mycelium on the thermal degradation of epoxy. Mycelium was selected as a representative bioderived flame retardant due to its proven ability to produce elevated char yield at temperatures above 325 °C^30,31^. Previous research conducted by our team^30^ revealed that alkaline-based deacetylation of mycelium can further increase char yields at high temperatures. Based on these findings, we developed mycelium-containing epoxy composites to enhance our understanding of the interaction between unmodified and deacetylated mycelium and epoxy resin, specifically the effects of mycelium derivatives on the thermal degradation behaviour of epoxy. We aimed to test the hypothesis that high char-forming mycelium (either unmodified or deacetylated) can improve the thermal stability of epoxy. To this end, we evaluated the performance of mycelium-containing epoxy composites in which the ratio of mycelium-to-epoxy was varied. This research will pave way for the integration of bioderived flame retardants into composite polymer matrices or flame-retardant coatings.

The outcomes of our research hold significant implications for fire sciences and fire engineering research communities. Firstly, this research holds the promise of providing insights into the influence of bioderived materials such as mycelium on the thermal decomposition of polymers, which can pave the way for sustainable fire safety solutions, improved fire performance, and reduced toxicity. Secondly, by reducing the reliance on chemically synthesized flame retardants, our research will contribute to a greener and more environmentally friendly approach to fire safety. Finally, our findings can help design mycelium-containing polymer coatings that can thermally protect flammable composites and minimise the release of toxic by-products during thermal decomposition. This new information has the potential to enhance flame retardancy, reduce smoke generation, and increase fire resistance, ultimately leading to the development of fire safe solutions for flammable materials.

## Materials and experiments

### Materials and mycelium cultivation

The selection of the non-pathogenic mycelium fungus, *Ganoderma australe*, for this study was based on its exceptional growth performance^31^ and relatively high chitin content in its cell wall^32^. A subculture of *Ganoderma australe*, provided by RMIT University’s Fungal Culture Collection (Australia), grown on malt extract agar was used as an inoculum for mycelium cultivation. The liquid feed solution, blackstrap molasses, was sourced from E&A Salce (Australia), while reagent-grade sodium hydroxide (NaOH) was obtained from Sigma Aldrich (Australia). An epoxy resin, diglycidyl ether of bisphenol A (DGEBA) (West System 105), and the corresponding slow-hardener (West System 206) were supplied by ATL Composites under the Gougeon Brothers Inc, (United States) licence.

Molasses feed solution (10 wt%) was prepared by diluting blackstrap molasses using deionized water. The feed solution was sterilised at 121 °C for 30 min to prevent bacterial contamination during mycelium incubation. Mycelium inoculum (6 mm diameter) was transferred to the dilute molasses solution (100 mL) in a sterilised polypropylene container. The culture was incubated in an environmental chamber under controlled temperature (~ 25 °C) and white light for 17 days. Mycelium sheets were harvested by peeling off the brownish gel at the feed/mycelium interface. Mycelium sheets were washed with water to remove molasses residue. Some mycelium sheets were deacetylated by submersion in 2 M NaOH at 40 °C for 8 h. Unmodified (control) and deacetylated mycelium samples are referred as the control and 2 M/40 °C/8 h, respectively. Deacetylation conditions, biomass yields, and the calculated degrees of deacetylation are given in Table 1. The degree of deacetylation was calculated in accordance with the FTIR-based calibration model reported in our previous study^30^. Control and deacetylated mycelium sheets were denatured at 60 °C for 3 h and kept in hermetic plastic bags to prevent moisture absorption during storage. Mycelium sheets were conditioned at 60 °C for 3 h before TGA experiments.

**Table 1:** Alkaline deacetylation conditions, biomass yields, degree of deacetylation, temperature at maximum mass loss rate, and remaining char at 600 and 850 °C during TGA experiments.

Sample	[NaOH] (M)	Temp (°C)	Time (hr)	Biomass (%)^a^	DD (%)^b^	*T*_max._ (°C)^c^	*Char*_600_ (%)^d^	*Char*_850_ (%)^e^
Control	–	–	–	100	45 ± 3	336 ± 5	36 ± 1	26 ± 0
2 M/40 °C/8 h	2	40	8	62 ± 3	84 ± 2	260 ± 6	48 ± 1	33 ± 2
Epoxy	–	–	–	–	–	419 ± 5	9 ± 1	7 ± 1

^a^Biomass (%) is the normalised dry mass yield of a deacetylated sample expressed as a percentage of the dry mass for the control unmodified mycelium.

^b^*DD* is the degree of deacetylation.

^c^*T*_max*.*_ is the temperature at the maximum rate of mass loss.

^d^*Char*_*600*_ and ^e^*Char*_*850*_ are char yields measured at 600 and 850 °C during TGA experiment expressed as a percentage of the initial sample mass.

### Manufacture of mycelium-only laminates and mycelium-containing epoxy composites

Flame retardants can be integrated into flammable composite laminates as thin interleaving layers, or micro-particulate additives to the polymer resins. To understand how the introduction of mycelium can influence the thermal degradation behaviour of an epoxy polymer matrix or coating, mycelium-containing epoxy composites were fabricated. Two layers of wet mycelium sheets (unmodified or deacetylated) were stacked and consolidated under elevated temperature (60 °C) and applied compression pressure (0.25 MPa) for at least 30 min to ensure complete drying. After cooling the two-ply mycelium composite to ambient temperatures, an epoxy/hardener slurry was uniformly applied on either side of the mycelium composite ensuring uniform resin spread across the whole surface. Mycelium-containing epoxy composites were cured at room temperature for 24 h and then post-cured at 60 °C for 8 h in a conventional oven.

**Figure 1:**
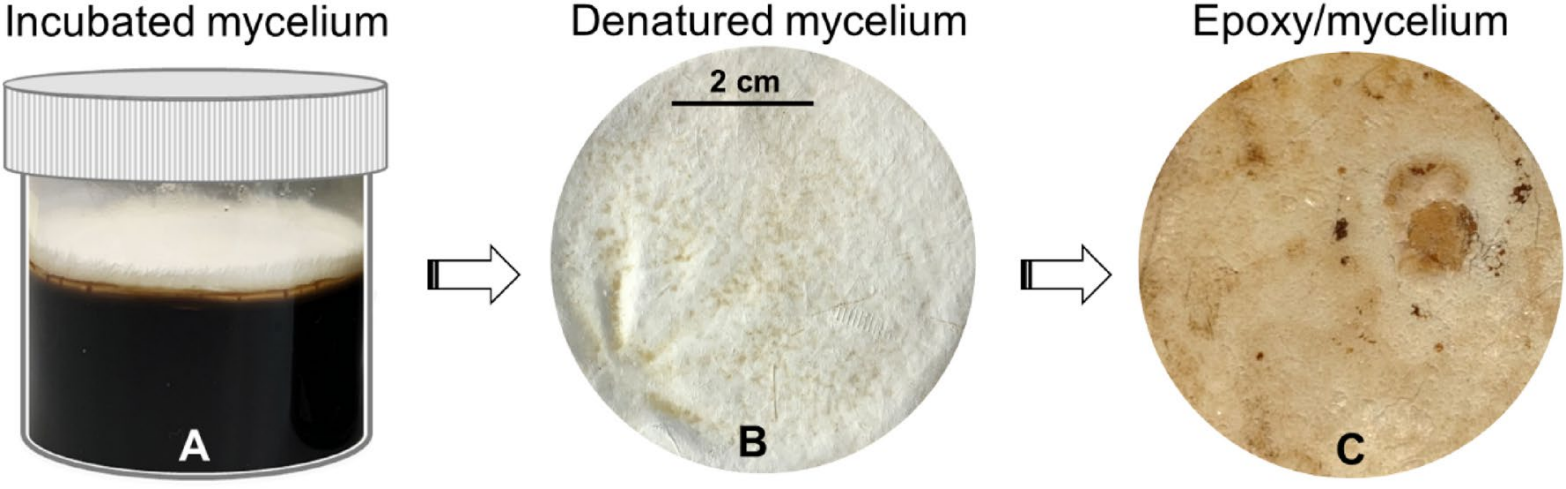
Photos of (**A**) incubated and (**B**) denatured mycelium, and (**C**) epoxy/mycelium composite.

Two sets of mycelium-containing epoxy composites were prepared. The first set of composites was used to investigate the effect of the mycelium-to-epoxy ratio on the thermal degradation behaviour of neat epoxy. Composites having mycelium-to-epoxy ratios of 1:2 (M1:E2), 1:1 (M1:E1) and 2:1 (M2:E1) were prepared using unmodified (control) mycelium. The same manufacturing methodology was followed in the preparation of the second set of composites in which the ratio of mycelium (control or deacetylated) to epoxy was fixed at 1:2. Composites in the second set are identified as epoxy/control and epoxy/2 M/40 °C/8 h. The post-cured mycelium-containing epoxy composites were shredded into small flakes and stored in hermetic plastic bags to prevent moisture absorption prior to TGA analysis. The composites were further dried at 60 °C for 3 h in a conventional oven just before conducting TGA experiments. The incubated and denatured mycelium, and the epoxy/mycelium composites are shown in Fig. 1.

Mycelium-only laminate specimens UL94 tests were manufactured by compression loading (0.25 MPa) wet mycelium sheets into 2 mm thick panels at 60 °C for at least 3 h. The consolidated mycelium-only laminates were cut into 125 × 13 × 2 mm specimens for UL-94 tests. Separately, mycelium-containing epoxy composite laminates in which the mycelium-to-epoxy ratio was 1:2, were manufactured for the UL-94 test. Epoxy-coated mycelium sheets were stacked to achieve a thickness of approximately 2.0 ± 0.2 mm. Separately, the neat epoxy sample was prepared by pouring the epoxy resin/hardener slurry into a flat-bottom rectangular container achieving a thickness of approximately 2 mm. The neat epoxy and mycelium-containing epoxy laminates were left to cure at room temperature for 24 h and subsequently post-cured at 60 °C for 8 h. The post-cured panels were cut into 125 × 13 × 2 mm specimens using a diamond saw. For the horizontal UL-94 tests, the post-cured specimens were marked at 25 and 100 mm from the free end where the flame was introduced.

### Thermogravimetric and evolved gas analysis (TGA-FTIR)

Changes to the thermal degradation behaviour epoxy in the presence of mycelium were investigated via thermogravimetric analysis using the Perkin Elmer STA 6000 TGA instrument interfaced with the Perkin Elmer Frontier FTIR Spectrometer for real-time chemical analysis of evolved gases. Flakes of test samples weighing ~ 12 mg were loaded into an alumina crucible which was then transferred into the TGA furnace. Initially, the furnace was set at 35 °C for 10 min to allow for sample equilibration. The furnace temperature was then increased at a rate of 30 °C/min until 850 °C under a continuous stream of N_2_ gas flowing at 20 mL/min. Some TGA experiments were stopped at 600 °C so that the primary residual char could be recovered for microstructural and FTIR analysis. An evolved gas transfer line connecting the TGA to the FTIR was maintained at 300 °C and continuously flushed with N_2_ gas (flow rate of 50 mL/min) to prevent condensation of evolved volatiles. Evolved gases were analysed between 4000 and 600/cm wavenumbers. A minimum of three repeat experiments were performed for each sample.

### Microstructural and morphological analysis of residual char

The FEI Quanta 200 scanning electron microscope was used for microstructural and morphological characterisation of both pristine mycelium and residual char recovered at 600 °C during TGA experiments. SEM specimens were attached to an electrically conductive carbon tape, which was then affixed to a sample holder. An iridium coating ~ 5 nm in thickness was applied onto the specimen using the Leica ACE 600 sputter coater, which was operated at 8 × 10^–3^ mbar. SEM images were collected using a spot size of 5 and accelerating voltages ranging between 15 and 30 kV depending on specimen thickness.

### Biochemical analysis of mycelium-containing epoxy composites before and after pyrolysis

The functional groups and chemical interaction between mycelium and epoxy were investigated using the Perkin Elmer (Spectra 100) FTIR Spectrometer equipped with an attenuated total reflection (ATR) lens at room temperature. Biochemical analysis was performed on mycelium-containing epoxy as well as the residual char recovered at 600 °C during TGA experiments. For each FTIR-ATR experiment, an average of 32 scans were recorded at a resolution of 4 cm^−1^ between 4000 and 600 cm^−1^. Absorption peaks were assigned using the built-in Perkin Elmer Spectrum 10.5.2 software. At least three repeat experiments were conducted for each sample.

### UL-94 vertical flammability and horizontal tests of mycelium-containing epoxy composites

The flammability of unmodified and deacetylated (2 M/40 °C/8 h) mycelium was assessed via the UL-94 vertical flame test, in accordance with ASTM D3801-20 guidelines^33^. During the UL-94 vertical test, the flame was applied for 10 ± 5 s and then removed. The flameout time was recorded as *t*_1_ in seconds. Once the specimen flamed out, the flame was immediately applied for the second time for about 10 s. After the flame was removed for the second time, the after-flame and afterglow times were respectively measured as *t*_21_ and *t*_3_, in seconds. Furthermore, the UL-94 horizontal flame test was performed to evaluate the flammability of neat epoxy, unmodified and deacetylated (2 M/40 °C/8 h) mycelium-containing epoxy composites, following ASTM D635-22 guidelines^34^. For the horizontal test, the flame was applied only once to each specimen, with a flame-impingement time of 30 ± 1 s. The time it took for the flame front to travel the 75 mm distance between the 25- and 100-mm reference markings on the test specimen was recorded. This data was then used to calculate the burning rate in mm/min. Five specimens were evaluated for each configuration.

## Results and discussion

### Thermal degradation characteristics of epoxy and mycelium

**Figure 2:**
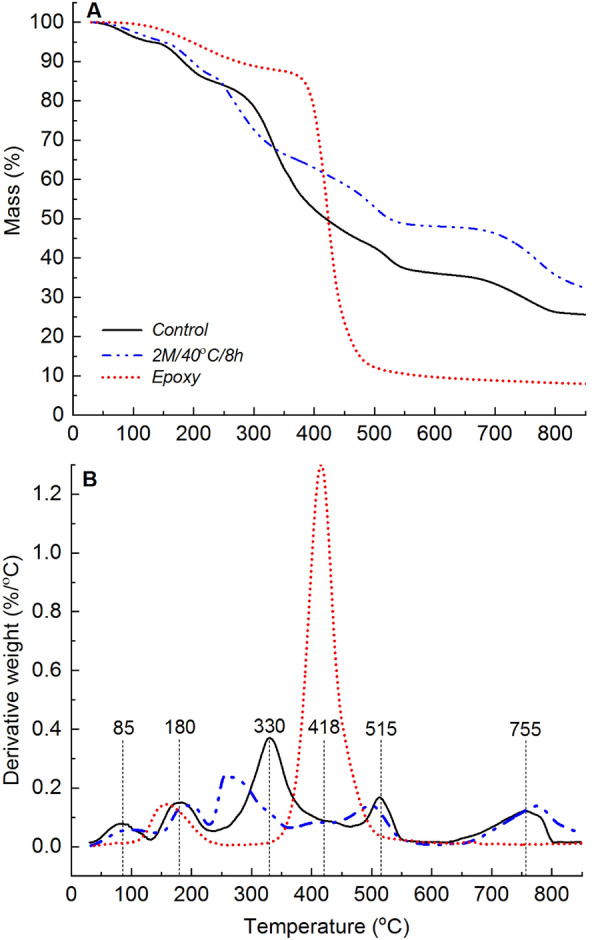
(**A**) TGA and (**B**) dTG profiles showing the thermal degradation behaviour of epoxy, and unmodified (control) and deacetylated (2 M/40 °C/8 h) mycelium.

The thermal degradation profile of neat epoxy consisted of two distinct mass loss events, as shown in Fig. 2. The first event, accounting for approximately 12% of the total mass loss, took place between 100 and 350 °C and was attributed to the loss of physically and chemically adsorbed water, as well as low molecular weight oligomers. The second event, which represented approximately 79% of the total mass loss, occurred between 350 and 600 °C and was associated with the main thermal decomposition of the epoxy polymer chains. During this secondary degradation step, epoxy underwent structural breakdown through chain scission, producing approximately 9% char yield at 600 °C. It is worth noting that at 850 °C, the recovered char constituted approximately 7% of the initial mass. This relatively low char yield indicates a significant degree of thermal-induced material volatilisation, with a relatively smaller fraction of the material ultimately converting into thermally stable carbonaceous char. The peak mass loss rate for the secondary thermal degradation step was approximately 418 °C as shown in Fig. 2B.

**Figure 3:**
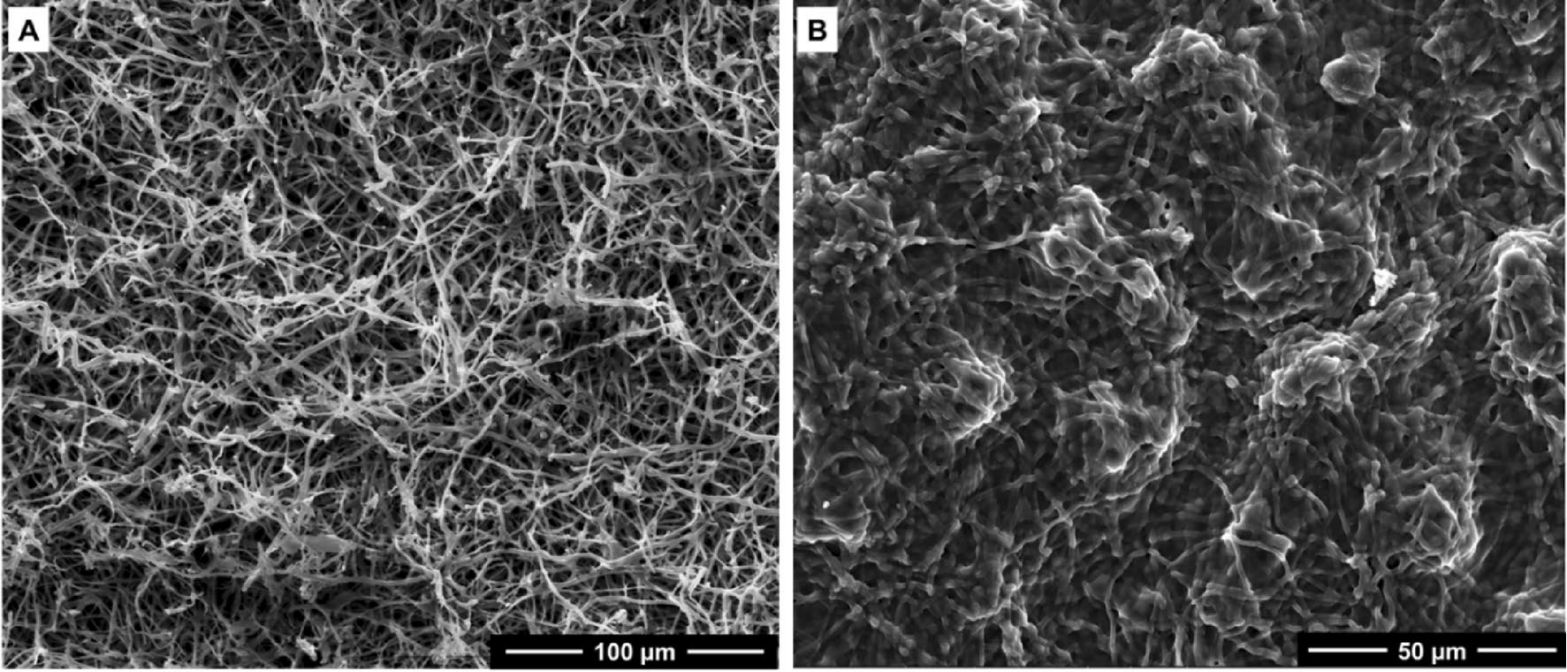
SEM images showing microstructural and morphological features of (**A**) unmodified (control) and (**B**) deacetylated (2 M/40 °C/8 h) mycelium.

In our previous study^30^, we reported that deacetylation of mycelium converted chitin into chitosan, which promoted char formation at high temperatures. However, we also observed that deacetylation reduced biomass yields. The reduction in biomass yields was attributed to deproteinisation, which involves the breaking of chemical bonds between chitin and proteins present in the cell wall of mycelium^35^. Consequently, deacetylated mycelium exhibited increased brittleness compared to unmodified mycelium^36^. Since a detailed discussion on this topic was presented in our previous work^30^, we provide a brief overview of the microstructural and morphological changes caused by deacetylation. Scanning electron microscopy images of unmodified (control) mycelium revealed a 3D hyphae filamentous network as shown in Fig. 3A. Alkaline deacetylation conditions promoted the fusion of hyphal filaments, resulting in the formation of micro-globular surface features and undulated surface morphologies, as shown in Fig. 3B for 2 M/40 °C/8 h.

Changes in the chemical, microstructural, and morphological features resulting from deacetylation can affect the thermal degradation behaviour of mycelium. The correlation between the degree of deacetylation and the thermal degradation behaviour of mycelium was extensively discussed in our previous study^30^. Key findings regarding the impact of deacetylation on the thermal degradation properties of mycelium, which provide the necessary background for this study, are briefly presented. Mass loss (TGA) and derivatised mass loss (dTG) profiles revealing five thermal degradation steps for both the control and deacetylated mycelium are shown in Fig. 2. The maximum mass loss rate events for the thermal degradation steps of unmodified mycelium were observed at 85, 180, 330, 515, and 755 °C. The maximum mass loss rate peaks for 2 M/40 °C/8 h were similar to those of the control, except for the third step, which occurred approximately 70 °C earlier at around 260 °C.

The thermal degradation behaviour of mycelium and its derivatives was characterised by several distinct mass loss steps. The first step, occurring between 35 and 130 °C, involved moisture desorption and the degradation and volatilisation of low molecular weight (< 20 kDa) hydrophobin fragments present on the surface of mycelium^37^. The second step, recorded between 130 and 235 °C, was associated with the loss of physically and chemically absorbed water molecules. The third step, occurring between 235 and 465 °C, was considered the primary thermal degradation step and attributed to the thermal decomposition of the fungal cell wall, which is composed of chitin, amino acids, carbohydrates (including polysaccharides), and glucan. The degradation of mycelium and its derivatives during this step involved deacetylation, depolymerisation, or cleavage of glycosidic linkages in the chitin segments of mycelium^38,39^. The fourth step, between 465 and 600 °C, formed primary char while the fifth and final step, occurring between 600 and 810 °C, involved the conversion of primary into secondary char. The char yields increased with the increase in the degree of deacetylation due to the superior thermal stability of chitosan compared to chitin. Additionally, the presence of low molecular weight OH-terminated units resulting from deacetylation promoted the formation of dense and consolidated char^40^.

**Figure 4:**
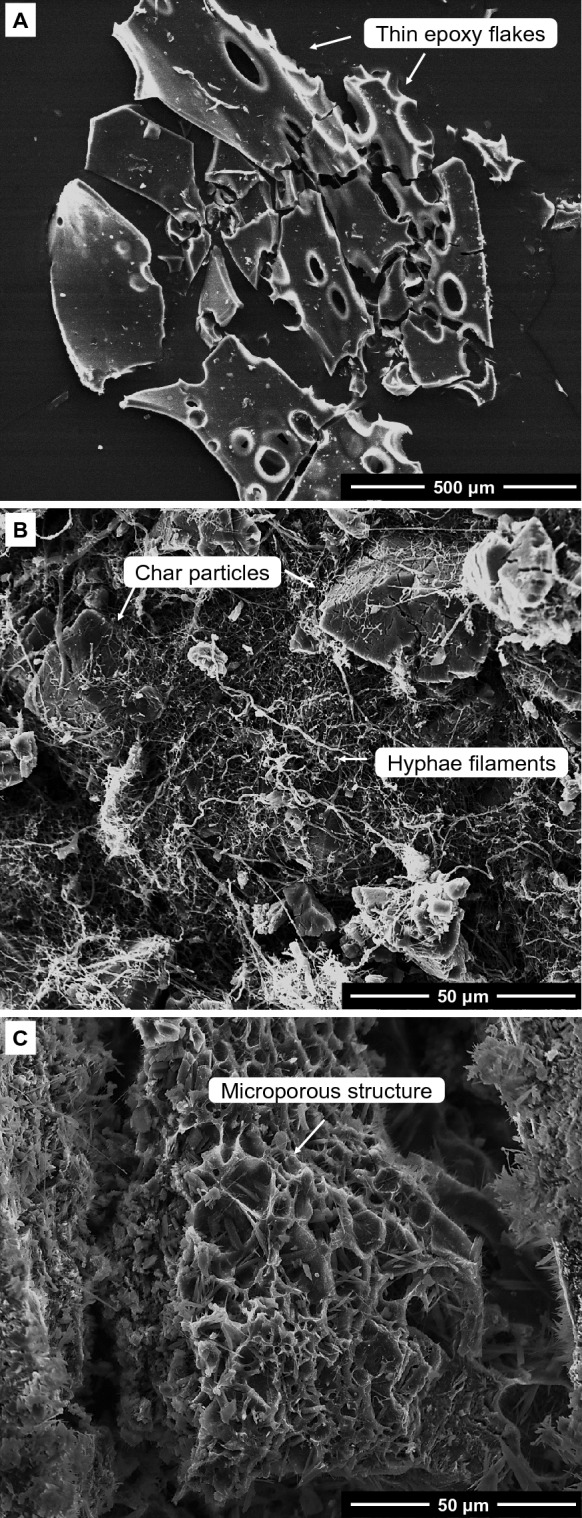
SEM image of char recovered at 600 °C for (**A**) epoxy, and (**B**) unmodified (control), and (**C**) deacetylated (2 M/40 °C/8 h) mycelium.

Figure 4 displays SEM images of the residual char obtained at 600 °C for epoxy, and control and deacetylated mycelium. The char produced by epoxy (Fig. 4A) appeared as thin, crystalline, and brittle flakes. In contrast, the char recovered from control mycelium (Fig. 4B) exhibited large particles with interspersed hyphae filaments. On the other hand, the char derived from deacetylated mycelium (Fig. 4C) displayed a distinct microstructure characterised by a microporous and rigid cellular structure. The presence of microporous and cellular microstructures in the char derived from 2 M/40 °C/8 h indicates that deacetylation not only increased the char but also created unique microstructural features such as the microporous network structure. Microporous and cellular structures can be effective in slowing down heat conduction due to the presence of voids or air-filled pores. To investigate the impact of char morphological variations between control and deacetylated mycelium on the thermal degradation of epoxy, thermogravimetric analysis was conducted on mycelium-containing epoxy composites.

### Effect of unmodified (control) mycelium on thermal degradation of epoxy

**Figure 5:**
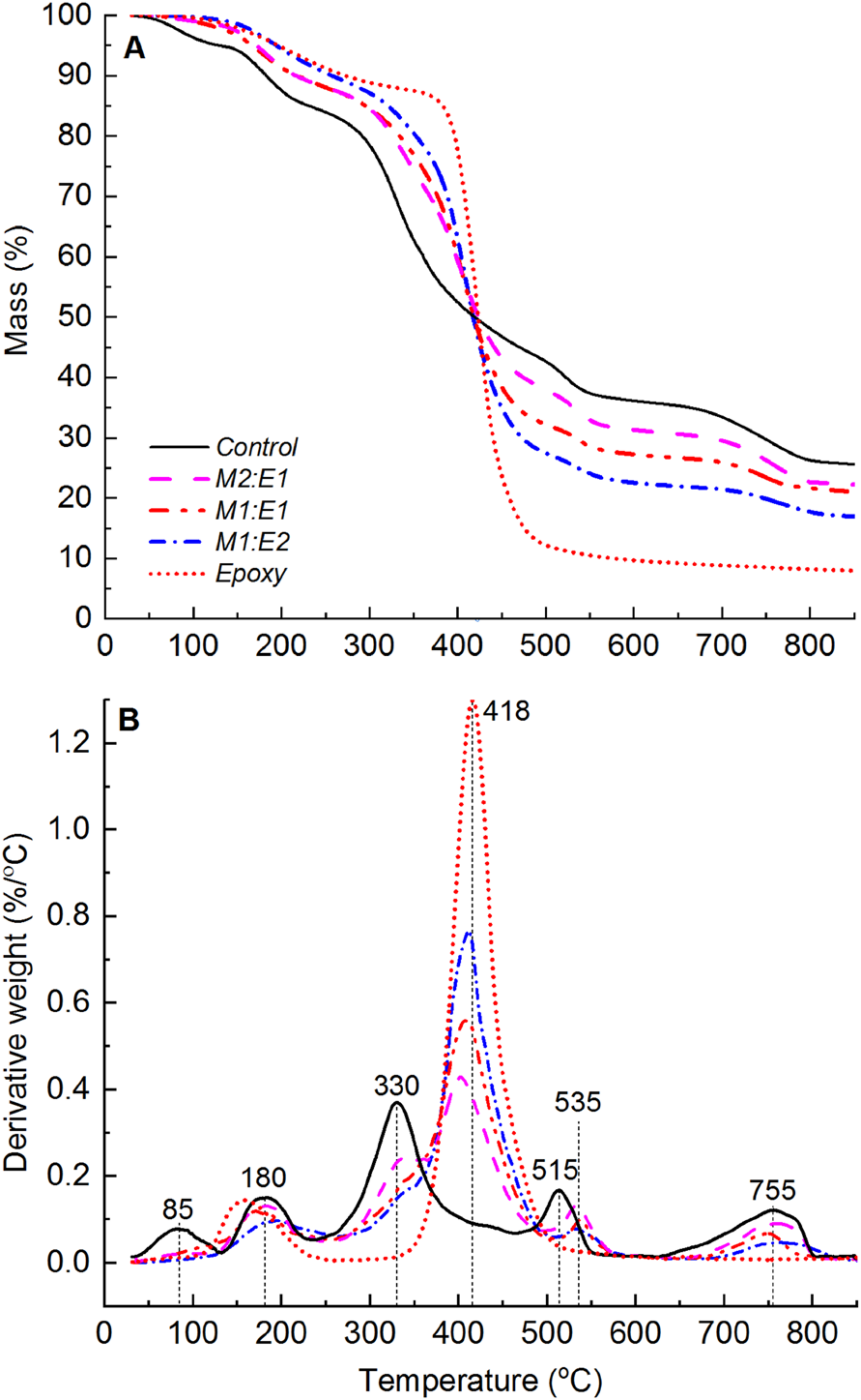
(**A**) TGA and (**B**) dTG profiles showing the thermal degradation behaviour of epoxy, unmodified (control) mycelium and mycelium-containing epoxy (M2:E1, M1:E1, and M1:E2) composites.

TGA experiments were performed to examine the effect of unmodified mycelium on the thermal degradation behaviour of epoxy. The TGA and dTG profiles are shown in Fig. 5, and the corresponding TGA data is presented in Table 2. The TGA mass loss profiles of mycelium-containing epoxy composites with varying mycelium-to-epoxy ratios were found to lie between those of neat epoxy and unmodified (control) mycelium. The dTG profiles of mycelium-containing epoxy exhibited five distinct mass loss events centred at 180, 330, 418, 535, and 755 °C. These mass loss events mirrored dTG peaks observed in unmodified mycelium and neat epoxy. However, there were substantial differences in material losses between 35 and 130 °C for mycelium-containing epoxy composites compared to unmodified mycelium. Specifically, dTG analysis revealed considerably lower mass losses within this temperature range for mycelium-containing epoxy composites, in contrast to the distinct dTG peak observed at 85 °C for unmodified mycelium. This suggests that the epoxy resin layer on the surface of mycelium acted as a physical barrier, hindering the volatilisation of low molecular weight hydrophobins. Another noteworthy distinction between unmodified mycelium and mycelium-containing epoxy composites was the shift in the fourth dTG peak. In the case of mycelium-containing epoxy composites, this peak was shifted by 20 °C (from 515 to 535 °C) relative to the unmodified mycelium. The shift to higher temperatures for the dTG peak suggests improved thermal stability for mycelium in the presence of epoxy. The improvement to the thermal stability of the primary char from mycelium in the presence of epoxy is not well understood and, therefore, requires further investigation.

**Table 2:** Composition of mycelium-containing epoxy composites, and temperature at maximum mass loss rate, T_max_ and char yields measured at 600 and 850 °C.

Sample	Mycelium (wt%)	Epoxy (wt%)	*T*_max._ (°C)^a^	*Char*_600_ (%)^b^	*Char*_850_ (%)^c^
Control	100	0	330 ± 2	36 ± 2	26 ± 1
M2:E1	67	33	405 ± 3	30 ± 2	21 ± 1
M1:E1	50	50	407 ± 3	28 ± 1	22 ± 1
M1:E2	33	67	411 ± 1	21 ± 1	16 ± 1
Epoxy	0	100	418 ± 4	9 ± 1	7 ± 1

^a^*T*_max_ is the temperature at maximum rate of mass loss and ^b^Char_600_ and ^c^Char_850_ are char yields measured at 600 and 850 °C during TGA experiments, respectively.

The dTG analysis of neat epoxy (Fig. 2) showed two distinct peaks representing material losses within the temperature ranges of 100–350 °C and 350–600 °C. The first peak, centred at 160 °C, was attributed to moisture desorption. Interestingly, this peak overlapped with the second peak observed for unmodified mycelium at 180 °C. In the case of the epoxy/control composite, prominent dTG peaks were observed with their peak values located between 405 and 411 °C. As the epoxy weight fraction decreased, the dTG peak height for mycelium-containing epoxy composites diminished. This suggests that the inclusion of unmodified mycelium causes a shift in the maximum mass loss temperature of epoxy towards lower temperatures. This shift is expected because mycelium has a relatively lower thermal degradation onset temperature. Interestingly, the char yields at temperatures above 418 °C increased with an increase in the mycelium-to-epoxy weight fraction ratio. This finding indicates that mycelium exhibits superior thermal stability compared to the neat epoxy resin at temperatures higher than 418 °C. In summary, the presence of unmodified mycelium reduced the maximum mass loss temperature, while simultaneously increasing the residual char yields at elevated temperatures. The increased surface residual char is a desirable characteristic of thermal protective coatings.

**Figure 6:**
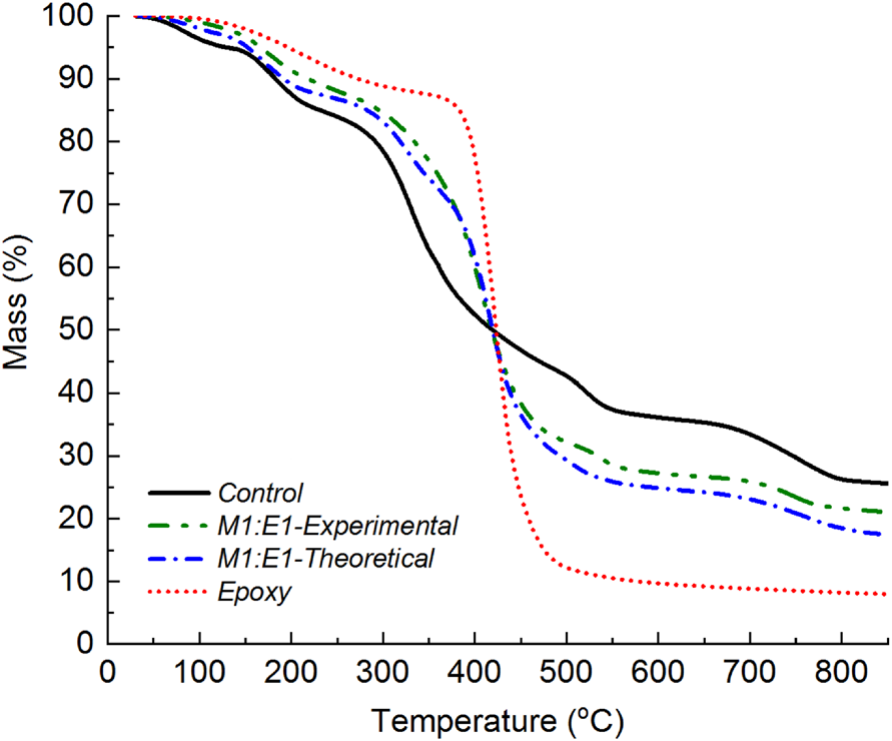
Experimentally measured and theoretically estimated TGA profiles for M1:E1 plotted alongside the mass loss profiles of control mycelium and epoxy.

The experimental TGA mass loss data for the M1:E1 ratio was compared with theoretically estimated data using an analytical approach based on fractional mass. The theoretical mass loss-temperature data for M1:E1 was calculated by summing the remaining mass fraction, $\alpha$, at specific temperatures according to Eq. (1):

1 $$\alpha = \sum_{i=1}^{p}\left({\alpha }_{i}{\gamma }_{i}\right),$$

where $i$ represents mycelium and epoxy components, $p$ is the total number of constituents which is two, ${\alpha }_{i}$ is the remaining mass fraction of either mycelium or epoxy at any given temperature calculated as the ratio between, the instantaneous mass at any given temperature and, the initial mass of the sample, and ${\gamma }_{i}$ is the weight percent of constituent $i$ at the beginning of the TGA experiment. Figure 6 demonstrates a strong correlation between the experimentally measured and theoretically estimated TGA profiles for the representative sample, M1:E1. This finding indicates that unmodified mycelium had a mere additive effect of the thermal degradation mechanism of epoxy. The improved char yields at high temperatures were due to the presence of the more thermally stable mycelium in mycelium-containing epoxy composites. On the other hand, the shift in the maximum mass loss temperature (*T*_*max*_) in mycelium-containing epoxy to a slightly lower value was caused by the early onset of mass loss in unmodified mycelium as revealed in Fig. 6 and summarised in Table 2. Other composites with different ratios of mycelium to epoxy (e.g., M2:E1 and M1:E2) showed similar trends between experimental and theoretical mass-temperature profiles.

**Figure 7:**
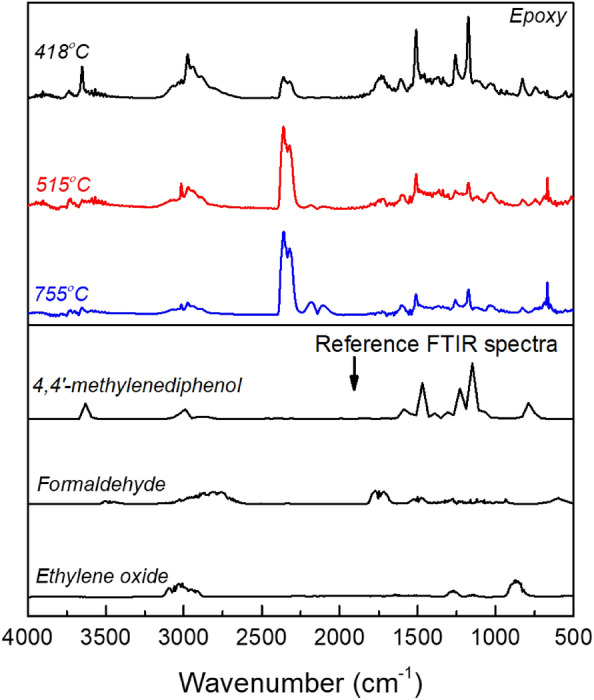
TGA-FTIR spectra of volatile gases released at 418, 515 and 755 °C during TGA pyrolysis of epoxy. Reference FTIR spectra for ethylene oxide, formaldehyde and 4,4′-methylenediphenol are included.

The analysis of evolved gases during the thermal degradation of TGA samples can provide valuable insights into potential changes in the degradation mechanisms of materials. In the case of neat epoxy, gases evolved at temperatures of 418, 515, and 755 °C were analysed using Fourier Transform Infrared (FTIR) spectroscopy, and the corresponding spectra are shown in Fig. 7. At 418 °C, the evolved gases included CO_2_ (~ 2349 and 669 cm^−1^)^41^, glyoxylic acid (~ 1595, 1383 and 1080 cm^−1^)^42^, ethylene oxide (~ 3057, 915 and 831cm^−1^)^43^, H_2_O (4000–3400 cm^−1^ and 1940–1277 cm^−1^)^44^, as well as a mixture of formaldehyde (~ 3401, 2928–2800 and 1710–1618 cm^−1^)^45^, and 4,4'-methylenediphenol (~ 1515, 1225 and 1173 cm^−1^)^46^. H_2_O is formed from a combination of hydroxy and hydrogen radicals, while CO_2_ is generated from the oxidative cleavage of the epoxy groups^1,47^. Similar evolved products were identified at higher temperatures of 515 and 755 °C, albeit with an increased relative proportion for CO_2_ and a reduced proportion for formaldehyde and 4,4′-methylenediphenol. Additionally, the presence of CO at ~ 2114 cm^−1^^41^ was observed at the higher temperature of 755 °C, indicating incomplete oxidation of the thermal degradation products under an inert atmosphere.

**Figure 8:**
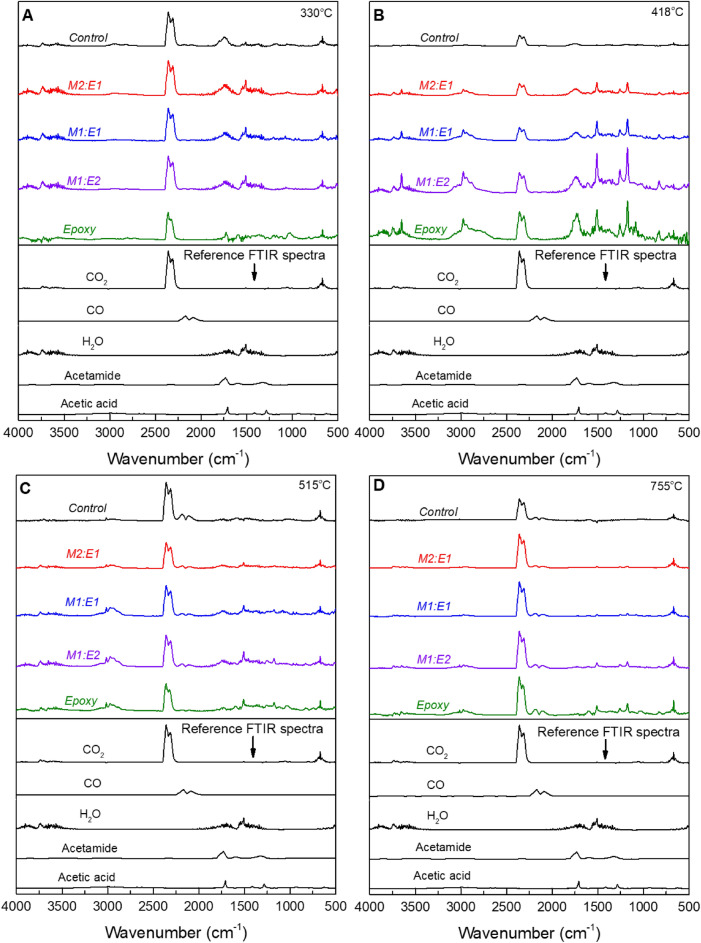
TGA-FTIR spectra for epoxy, and control and mycelium-containing epoxy composites (M1:E2; M1:E1; M2:E1) recorded at TGA temperatures of (**A**) 330, (**B**) 418, (**C**) 515 and (**D**) 755 °C.

Figure 8 displays the TGA-FTIR spectra of mycelium-containing epoxy composites, plotted together with unmodified mycelium and neat epoxy. Interestingly, the same absorption patterns observed in the FTIR spectra of volatiles produced by the neat epoxy were identified in all mycelium-containing epoxy composites. The intensity of the FTIR peaks associated with the volatiles released during the thermal decomposition of epoxy decreased as the mycelium-to-epoxy ratio increased. This trend indicates that the influence of epoxy diminishes as the weight fraction of mycelium in the mycelium-containing epoxy composites increases. FTIR peaks observed at 330 °C, were due to the decomposition volatiles of mycelium. The distinctive and intense FTIR peaks in the range 1750–1780 cm^−1^ were identified as the aldehyde carbonyl group produced during the thermal decomposition of mycelium^48^. Furthermore, the sharp peaks at approximately 1591 and 1512 cm^−1^ are characteristic of aromatic heterocyclic compounds formed during the thermal degradation of mycelium^48^. At 418 °C, the volatiles released from mycelium-containing epoxy had similar FTIR spectra to neat epoxy. Similar patterns were observed at 515 and 755 °C, where the gaseous thermal decomposition products of mycelium-containing epoxy composites exhibited the same characteristic features as those reported for neat epoxy. These findings provide insights into the volatiles released during the thermal degradation of mycelium-containing epoxy and more important they suggest subtle changes to the thermal degradation pathway of epoxy due to the presence of mycelium especially when considering evolved gases.

**Figure 9:**
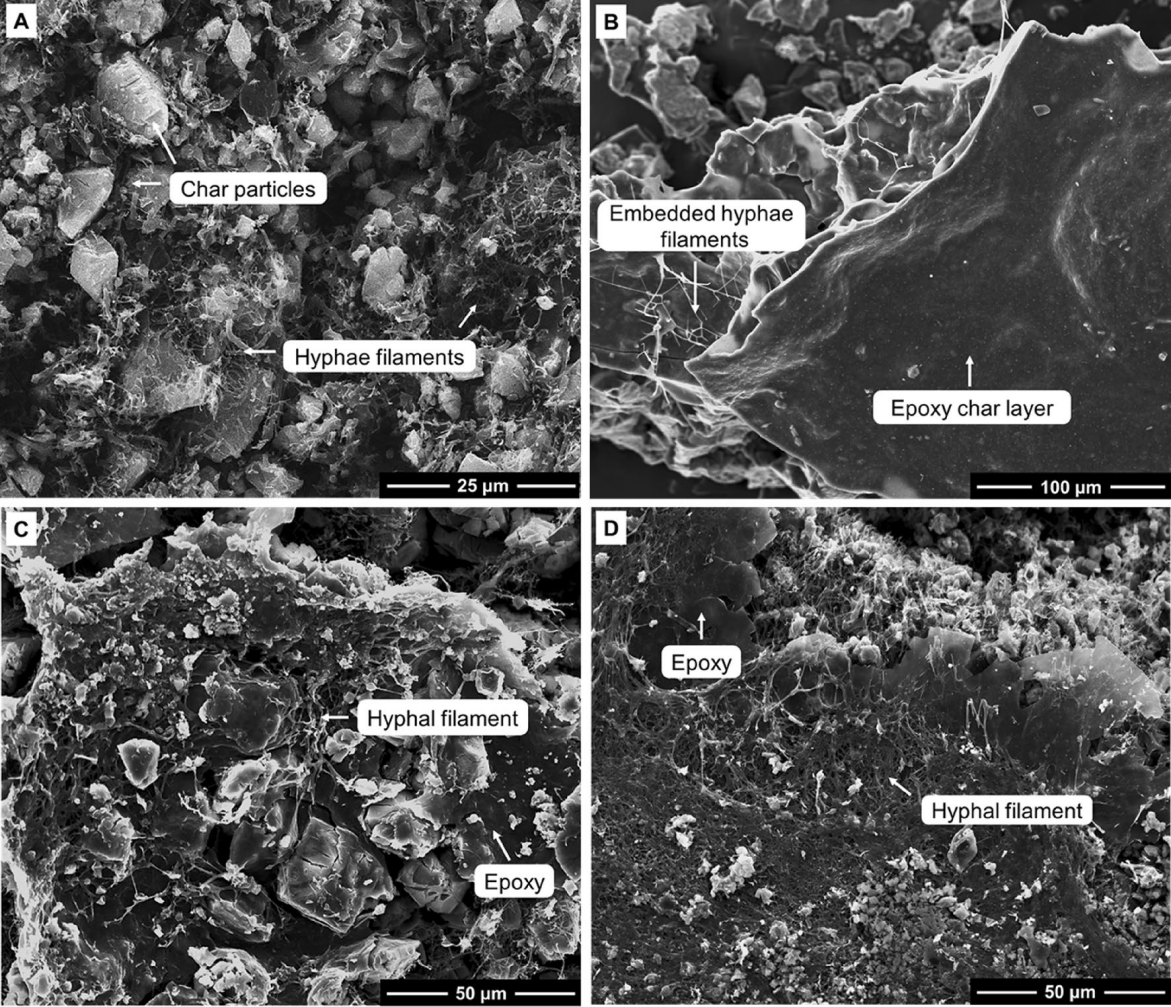
SEM image of char recovered at 600 °C during TGA experiments for the (**A**) control, and (**B**) M1:E2, (**C**) M1:E1 and (**D**) M2:E1 composites.

To investigate the relationship between char morphology and the thermal degradation behaviour of unmodified mycelium and mycelium-containing epoxy, char material recovered at 600 °C were analysed using the scanning electron microscopy. Figure 9 shows the SEM images of residual char from unmodified mycelium and its mycelium-containing epoxy counterparts. The char obtained from unmodified mycelium displayed remnants of a three-dimensional hyphae filamentous network, along with micro-sized char particles (Fig. 9A). In the residual char of mycelium-containing epoxy composites, hyphae filaments were visible, and their density increased as the mycelium-to-epoxy weight ratio increased. The composite with the highest epoxy resin content, M1:E2, revealed crystalline and continuous char particles with embedded hyphae filaments as shown in Fig. 9B. As the mycelium content increased, the prominence of crystalline char particles decreased (Fig. 9C,D), and the char morphology resembled that of unmodified mycelium (Fig. 4B). Although there were noticeable variations in char morphology with changes in the mycelium-to-epoxy ratio, this analysis did not provide insights into the relationship between char characteristics and the thermal stability of mycelium-containing epoxy composites. Therefore, FTIR spectroscopic analysis of the residual char was conducted to improve our understanding of the degradation behaviour of mycelium-containing epoxy composites.

**Figure 10:**
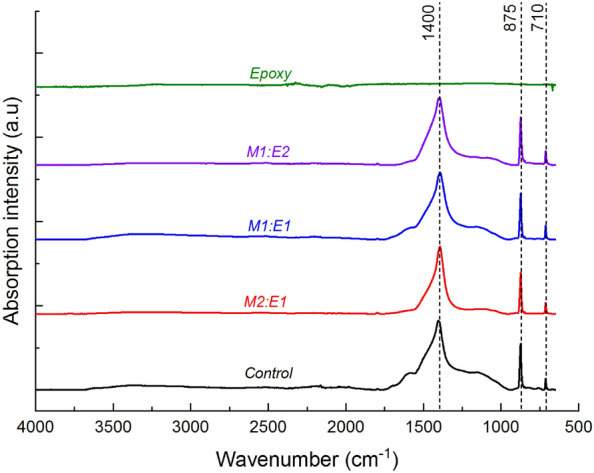
ATR-FTIR spectra of char material recovered at 600 °C during TGA experiments for epoxy, unmodified (control) mycelium and mycelium-containing epoxy composites.

Figure 10 presents the ATR-FTIR spectra of residual char obtained from unmodified mycelium and mycelium-containing epoxy composites at 600 °C during TGA experiments. The primary residual char from both unmodified mycelium and mycelium-containing epoxy composites exhibited a prominent and intense absorption peak at approximately 1400 cm^−1^, which was attributed to C–H bending or aromatic C=C bonds^49^. Remarkably, this absorption peak persisted in the residual char recovered at 850 °C (spectra not shown here), suggesting its association with aromatic C=C bonds. Additionally, another distinct and moderately strong absorption peak, centred around 875 cm^−1^, was observed in the FTIR spectra of char recovered at 600 °C for both unmodified mycelium and mycelium-containing epoxy composites. This absorption band was assigned to C–H bending of the furanose ring^50,51^. Furanose, is a product of ring-chain tautomerism of pyranose at high temperatures and is likely present in the recovered char. Furthermore, a weak yet sharp peak at approximately 710 cm^−1^ appeared in both the char derived from unmodified mycelium and mycelium-containing epoxy, which was attributed to aromatic ring C–H bending^49^. The similarity in FTIR spectroscopic features between unmodified mycelium and its mycelium-containing epoxy counterparts suggests the absence of chemical interactions between mycelium and epoxy, as well as their thermal degradation products.

In essence, the presence of unmodified mycelium did not substantially affect the thermal degradation behaviour of neat epoxy, as indicated by the analysis of evolved gases and FTIR characterisation of the recovered char. The thermally degradation of epoxy preceded the formation of primary char from the thermal decomposition of mycelium. By the time mycelium formed the primary char, the epoxy resin component had already been depleted. These findings suggest that the by-products of the thermal decomposition of mycelium at temperatures below 418 °C have very little impact on the primary pyrolysis mechanism of epoxy. While mycelium may not alter the thermal degradation pathways of epoxy, its ability to promote char formation when incorporated into polymer matrices can be harnessed to create fireproofing coatings for flammable composites. When exposed to high temperatures, mycelium-containing epoxy coatings will rapidly generate a consolidated char layer on the surface of a flammable substrate. The physical char layer then serves to reduce heat transfer to the flammable substrate and prevent the escape of pyrolysis products into the combustion zone thereby limiting combustion fuel.

### Effect of deacetylated mycelium on the thermal degradation of epoxy

In our previous studies, we reported that deacetylated mycelium generated higher char yields than its unmodified counterpart^30^. The increased char formation was attributed to the presence of more thermally stable chitosan in deacetylated mycelium. Furthermore, deacetylation produced hydroxyl-group terminated low molecular weight polysaccharides which have been shown to promote char formation^48^. In this study, we investigated whether the enhanced char formation in deacetylated mycelium would have different effects on the thermal degradation behaviour of epoxy compared to unmodified mycelium. A deacetylated mycelium-containing epoxy composite (epoxy/2 M/40 °C/8 h) with a mycelium-to-epoxy ratio of 1:2 ratio was prepared. Typically, fire retardant additives are integrated into polymers at loading fractions lower than 40 wt% to preclude processing issues and mechanical property degradation of the resultant composite^23^.

**Figure 11:**
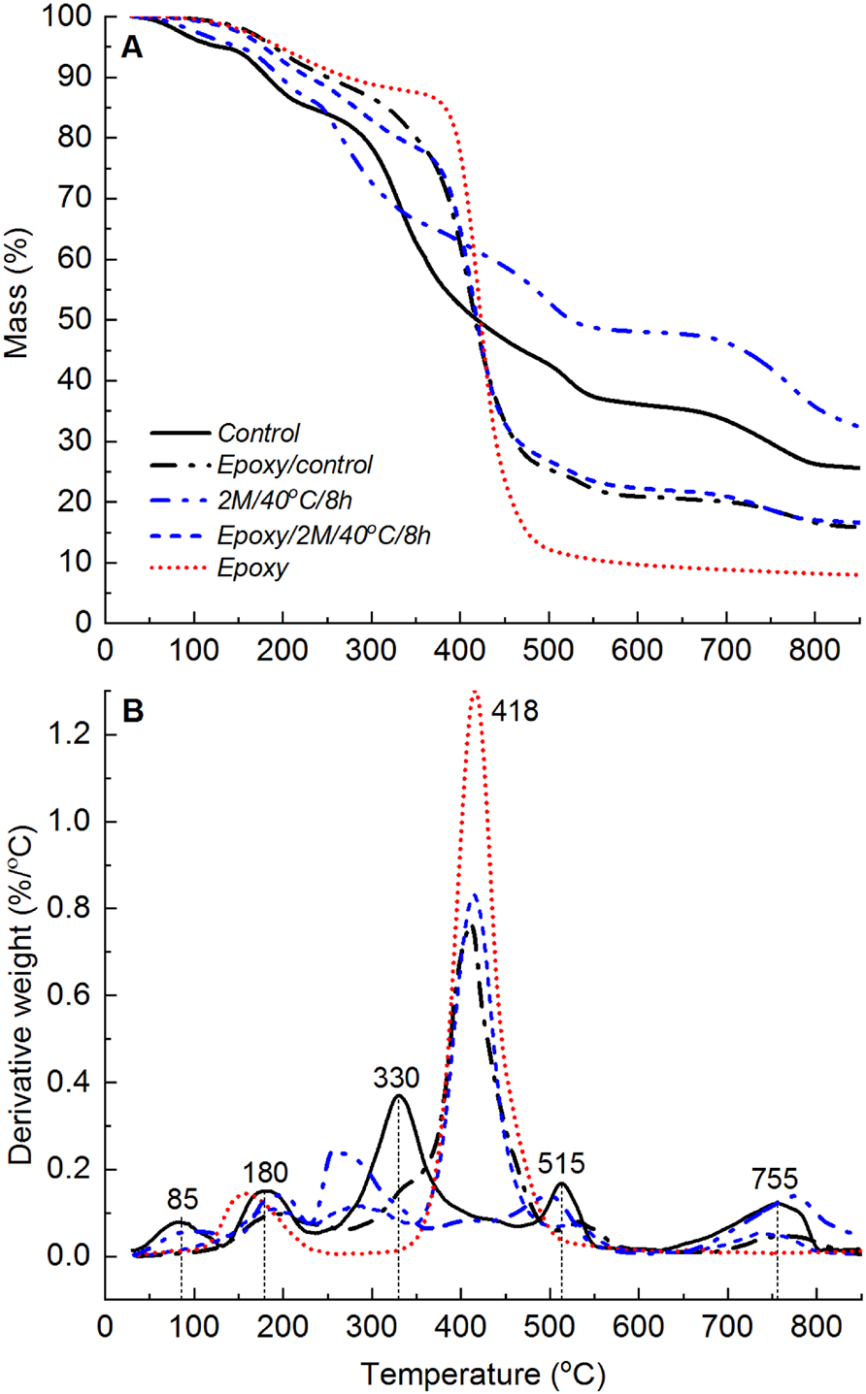
(**A**) TGA and (**B**) dTG curves showing the thermal degradation profiles of the unmodified (control) mycelium, epoxy/control, 2 M/40 °C/8 h, epoxy/2 M/40 °C/8 h and neat epoxy.

**Figure 12:**
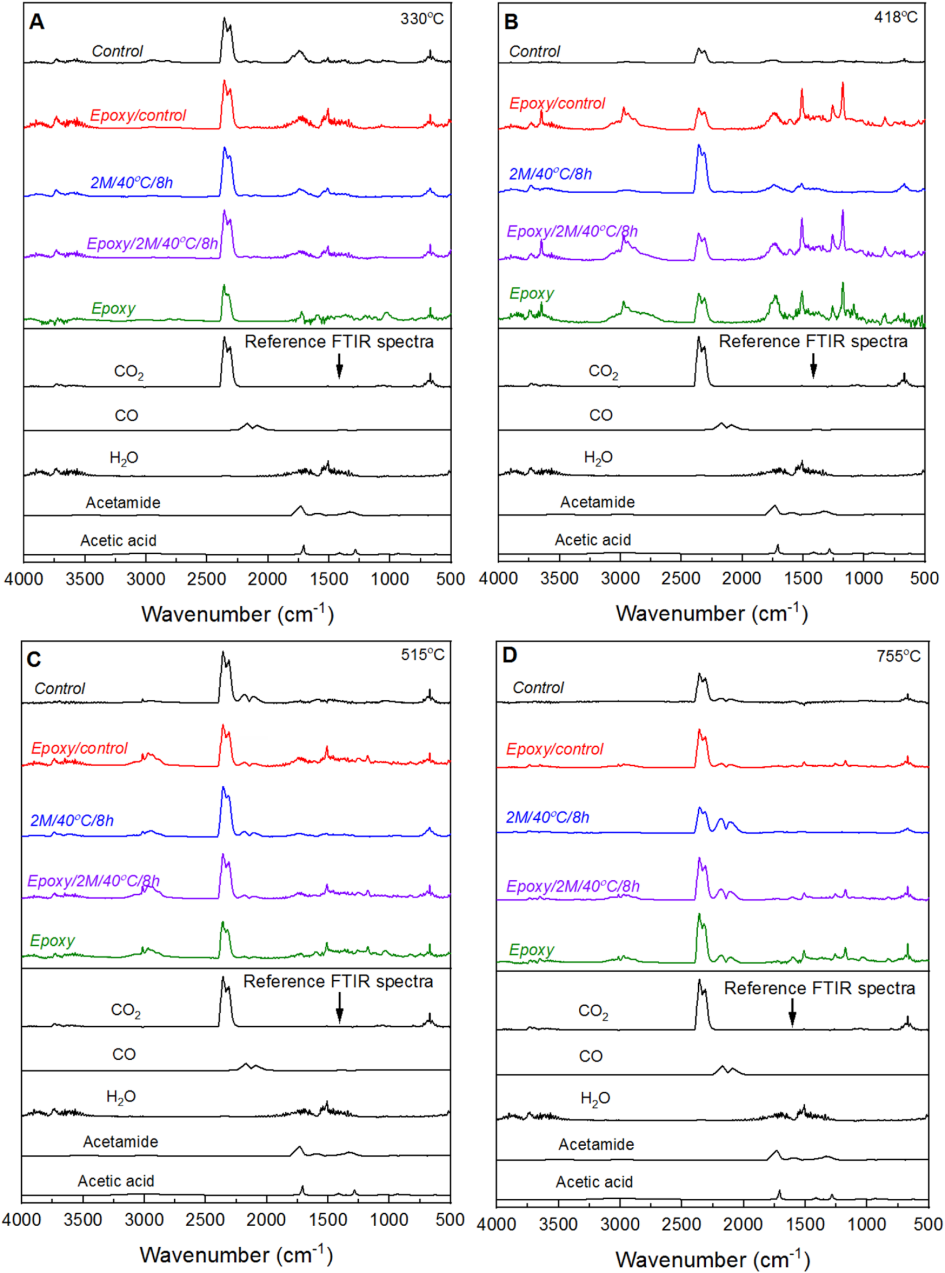
TGA-FTIR spectra for unmodified (control) mycelium, 2 M/40 °C/8 h, epoxy/control, epoxy/2 M/40 °C/8 h and neat epoxy recorded at TGA temperatures of (**A**) 330, (**B**) 418, (**C**) 515 and (**D**) 755 °C.

**Figure 13:**
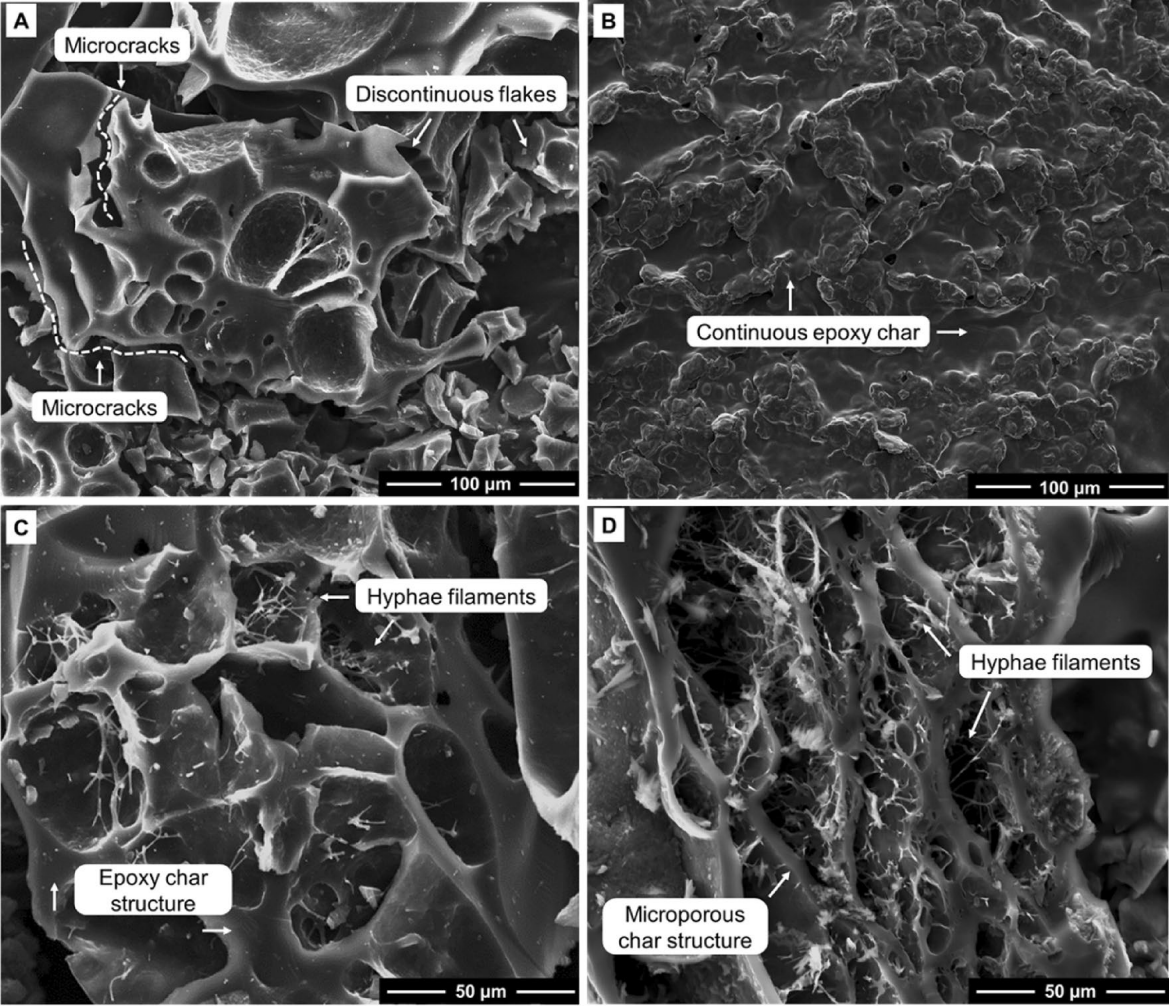
Low and high-magnification SEM images of char recovered at 600 °C for (**A,C**) epoxy/control and (**B,D**) epoxy/2 M/40 °C/8 h composites.

Figure 11 presents the TGA mass loss and corresponding dTG profiles of epoxy/control, and epoxy/2 M/40 °C/8h composites, along with the mass loss curves for unmodified (control) mycelium, 2 M/40 °C/8 h, and neat epoxy. The extracted TGA data is presented in Table 3. The char yield from the epoxy/2 M/40 °C/8 h composite was similar to that from the epoxy/control composite suggesting limited changes to the thermal degradation behaviour of epoxy due to increased degree of deacetylation. TGA-FTIR analysis conducted at 330, 418, 515 and 755 °C (Fig. 12) revealed that the gaseous products generated from the pyrolysis of mycelium-containing epoxy composites were primarily due to the thermal degradation of epoxy. The FTIR spectra showed no differences between epoxy/control and epoxy/2 M/40 °C/8 h composites. This indicates that, while deacetylated mycelium produces more char, like unmodified mycelium, it has little effect on the thermal degradation pathway of epoxy. Microscopic characterisation of the residual char recovered at 600 °C was conducted to gain an in-depth understanding of the differences in thermal degradation pathways of the epoxy/control and epoxy/2 M/40 °C/8 h composites. Figure 13 presents SEM images of the char residue from epoxy/control and epoxy/2 M/40 °C/8 h composites revealing hyphae filaments embedded within char structures. The solid residue from the epoxy/control composite exhibited microcracked and discontinuous flake-like char particles. The char from the epoxy/2 M/40 °C/8 h composite displayed a continuous layer which was likely the residue from the degradation of epoxy covering the residue of deacetylated mycelium. Despite the presence of epoxy, the microstructure of the char recovered from the epoxy/2 M/40 °C/8 h composite was similar to that of 2 M/40 °C/8 h, as presented in Fig. 4C.

**Table 3:** Composition of mycelium-containing epoxy composites, temperature at maximum mass loss rate, T_max_ and char yields measured at 600 and 850 °C.

Sample	Mycelium (wt%)	Epoxy (wt%)	*T*_max._ (°C)^a^	*Char*_600_ (%)^b^	*Char*_850_ (%)^c^
Control	100	0	330 ± 2	36 ± 2	26 ± 1
2 M/40 °C/8 h	100	0	260 ± 6	48 ± 1	33 ± 2
Epoxy/control	33	67	411 ± 1	21 ± 1	16 ± 1
Epoxy/2 M/40 °C/8 h	33	67	413 ± 2	22 ± 3	17 ± 2
Epoxy	0	100	418 ± 4	9 ± 1	7 ± 1

^a^*T*_max_ is the temperature at maximum rate of mass loss and ^b^Char_600_ and ^c^Char_850_ are char yields measured at 600 and 850 °C during TGA experiments, respectively.

**Figure 14:**
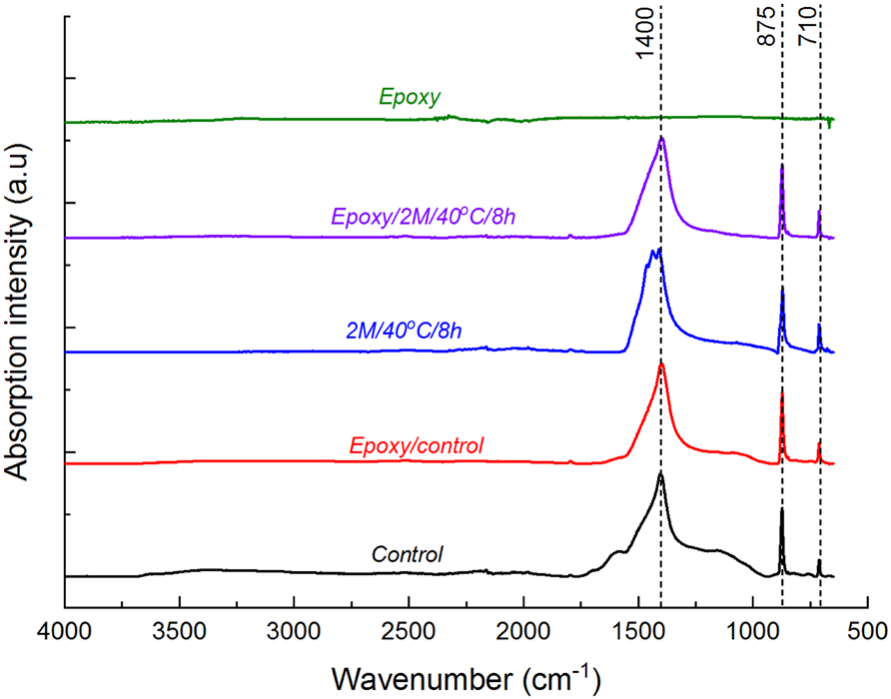
ATR-FTIR spectra of char recovered at 600 °C during TGA experiments for epoxy, unmodified (control) mycelium, 2 M/40 °C/8 h, and epoxy/control and epoxy/2 M/40 °C/8 h.

Interestingly, the ATR-FTIR analysis of the char recovered from the epoxy/control and epoxy/2 M/40 °C/8 h composites revealed identical spectroscopic features as shown in Fig. 14. The residual char primarily consisted of degradation products from deacetylated mycelium, without evidence of the epoxy residue. These findings, like in the case of unmodified mycelium, suggest that the presence of deacetylated mycelium has little influence on the thermal degradation pathways of epoxy apart from improving the residual char yields. In summary, incorporating deacetylated mycelium directly into epoxy resins has minimal effect on the thermal degradation pathways of polymer matrix but, like unmodified mycelium, increases char yields. Thus deacetylated mycelium-containing epoxy formulations can be employed as sacrificial surface thermal protection materials for flammable substrates, as demonstrated in our earlier work^52^. However, if the mycelium/epoxy formulations were to be used for thermal surface protection, it would be critical to evaluate their flammability or flame spread characteristics.

UL-94 flammability tests were performed to assess the fire safety characteristics of mycelium and mycelium-containing epoxy composites. Both vertical and horizontal configurations of UL-94 flammability tests were conducted to measure the rate of fire propagation and identify whether the material promotes or inhibits flame spread. Parameters such as the time of flame persistence, dripping behaviour, and burning rate were measured.

Under the vertical test mode, the V-rating of unmodified, deacetylated mycelium, and mycelium-containing epoxy composites are presented in Table 4, showcasing the after-flame time, afterglow time, and dripping behaviour of the specimens. The term “after-flame time” refers to the duration of continued burning after the ignition source has been removed, while “afterglow time” indicates the duration of smouldering. The highest V-rating, V-0, signifies materials with the lowest flammability and where there is no occurrence of flammable drips. The results in Table 4 showed that unmodified mycelium obtained a V-1 rating, while deacetylated (2 M/40 °C/8 h) mycelium achieved the highest V-0 rating.

**Table 4:** UL-94 vertical classification of unmodified (control) and deacetylated mycelium and mycelium-containing epoxy composites.

Sample	*t*_1_ (s)	(*t*_1_ + *t*_2_) (s)^a^	(*t*_2_ + *t*_3_) (s)^b^	Combustion up to clamp^c^	Dripping specimens^d^	UL-94 V-rating
Control	13	150	57	No	No	V-1
2 M/40 °C/8 h	1	14	9	No	No	V-0
Epoxy/control	N/A	N/A	N/A	Yes	No	No rating
Epoxy/2 M/40 °C/8 h	N/A	N/A	N/A	Yes	No	No rating
Epoxy	N/A	N/A	N/A	Yes	Yes	No rating

*t*_1_ is after-flame time after the first flame application in second; *t*_*2*_ is after-flame time after the second flame application in second; *t*_*3*_ is afterglow times after the second flame application in second.

^a^(*t*_1_ + *t*_2_) is the total summation of after-flame time for any condition set of five specimens.

^b^*(t*_*2*_ + *t*_*3*_*)* presents the average of after-flame plus afterglow time for each individual specimen after second flame application in second.

^c^Combustion up to holding clamp indicates that the specimen was completely burned.

^d^Dripping of burning specimens which ignites the underneath cotton batting.

**Figure 15:**
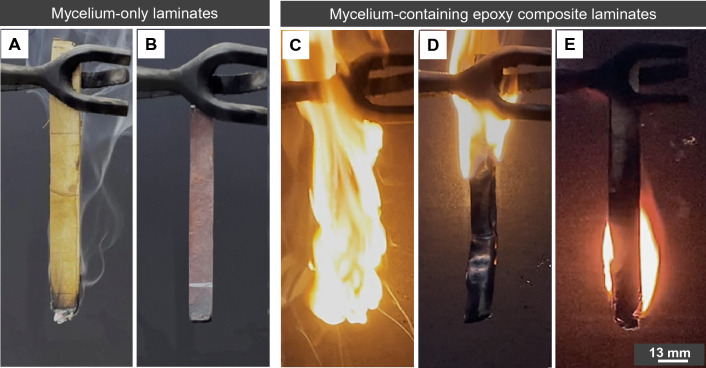
Images captured from the UL-94 vertical test at 35 s after ignition source had been removed for (**A**) unmodified (control), (**B**) 2 M/40 °C/8 h, (**C**) epoxy, (**D**) epoxy/control and (**E**) epoxy/2 M/40 °C/8 h.

Figure 15 presents captured images 35 s after the ignition source had been removed. In the case of unmodified mycelium, the flame persisted for 13 s after the ignition source was removed, leaving behind a smouldering specimen. In contrast, deacetylated mycelium flamed for less than 1 s before the flame extinguished completely, without any lingering smouldering. Notably, deacetylated mycelium, with its V-0 rating, exhibited a high level of fire resistance, showcasing outstanding performance according to the UL-94 flammability test. These findings suggest that deacetylated mycelium possessed superior fire-retardant properties and higher resistance to flame propagation compared to its unmodified counterpart. However, both the epoxy and mycelium-containing epoxy composites failed to pass vertical test due to exceeding the after-flame time limit (*t*_1_ ≥ 30 s). Although the presence of mycelium did not lead to a V-rating under vertical UL94 conditions, it did alter the burning behaviour of neat epoxy. While epoxy dripped and ignited the cotton batting during combustion, composites containing both unmodified and deacetylated mycelium did not exhibit dripping. Furthermore, the difference in burning rate between epoxy and mycelium-containing epoxy composites was noticeable, as reflected in Fig. 15C–E. Subsequently, the flammability of mycelium-containing epoxy composites was evaluated under the horizontal configuration and compared to the neat epoxy.

**Figure 16:**
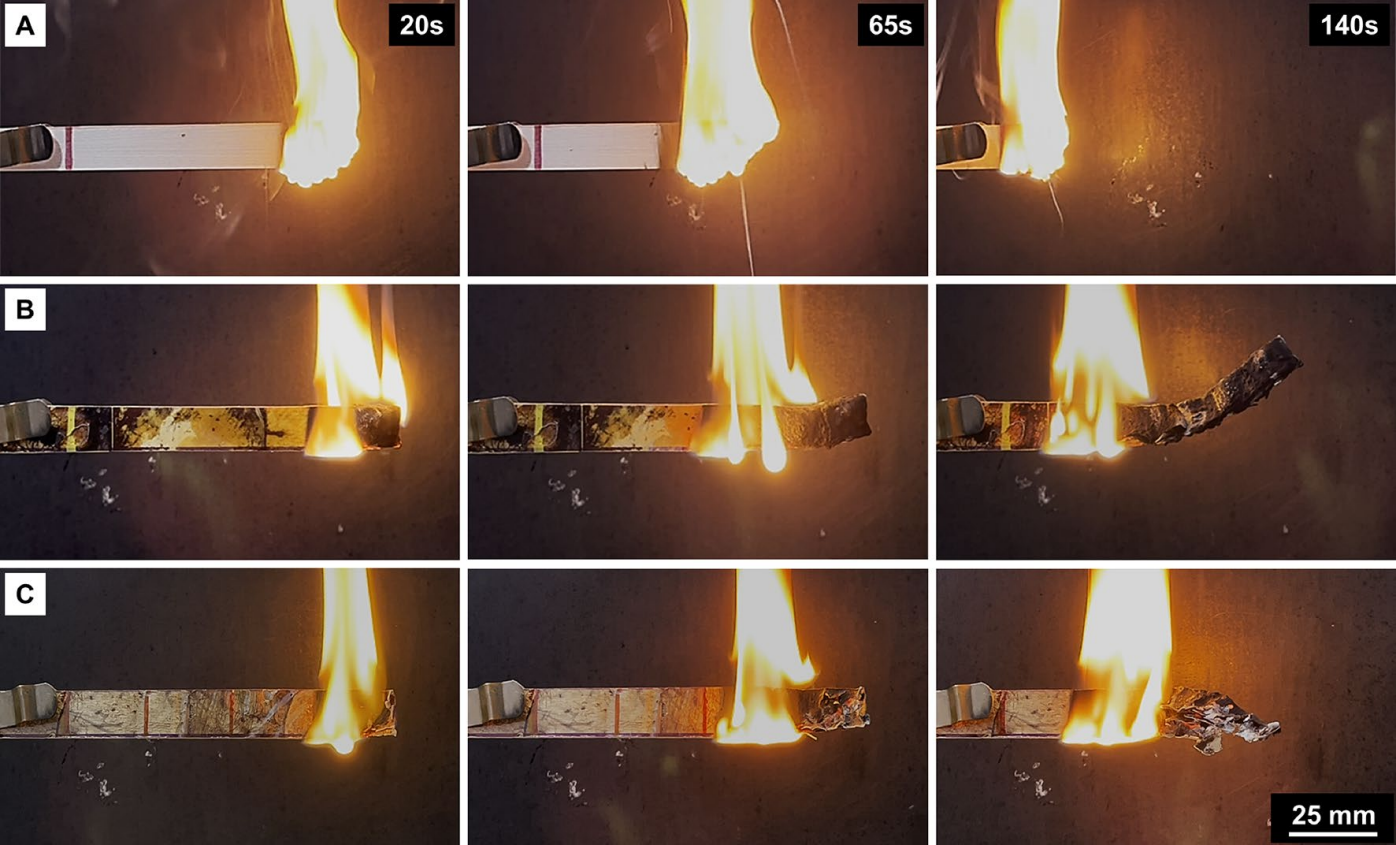
Images captured from the UL-94 horizontal test for (**A**) epoxy, (**B**) epoxy/control and (**C**) epoxy/2 M/40 °C/8 h at 20, 65 and 140 s after the ignition source had been removed. The images at 20 and 140 s correspond to the flame front in the neat epoxy specimen reaching 25 mm and 100 mm markings along the specimen length, respectively.

The experimental results obtained from UL-94 horizontal test are reported in Table 5 and presented in Fig. 16. Both the neat epoxy and mycelium-containing epoxy composites achieved the HB rating since their burning rate was below 75 mm/min. Despite all three specimens achieving the HB rating, it is noteworthy, the burning rate for the neat epoxy was higher than that measured for mycelium-containing epoxy composites. In fact, the burning rate for the epoxy/control and epoxy/2 M/40* °C*/8 h samples were 9% and 29% respectively slower than the neat epoxy. That is, the flame spread in mycelium-containing epoxy composites was supressed suggesting improved fire safety ratings in comparison to the neat polymer. The superior fire safety ranking of mycelium-containing epoxy composites was achieved despite a relatively high weight fraction of the highly flammable epoxy polymer. Thus, if mycelium were to be used as fireproofing material with minimal polymer binder content, it would be expected to perform even better. In summary, this research has demonstrated the feasibility of developing mycelium-based fireproofing materials with improved fire safety for flammable substrates including flammable polymer matrix composites.

**Table 5:** UL-94 horizontal classification of mycelium-containing epoxy composites and neat epoxy.

Sample	Elapsed time (s)	Burning rate (mm/min)	UL-94 HB rating
Epoxy	128 ± 4	35 ± 1	HB
Epoxy/control	140 ± 4	32 ± 1	HB
Epoxy/2 M/40 °C/8 h	173 ± 7	25 ± 2	HB

## Conclusions

This study investigated the influence of mycelium, both unmodified and deacetylated, on the thermal degradation behaviour and char-forming characteristics of epoxy matrix composite formulations. The key findings can be summarised as follows:

- The inclusion of mycelium in epoxy resulted in a shift in the maximum mass loss rate temperature from 418 °C for the neat epoxy to a range of 405–411 °C for the mycelium-containing epoxy composites. This reduction in temperature was attributed to the early onset of thermal degradation caused by the presence of mycelium.
- No apparent synergistic effects were observed due to the presence of mycelium in epoxy, apart from the shift in the peak temperature (515 to 535 °C) for the conversion of primary into secondary char.
- Primary and secondary char formation by mycelium occurred after the epoxy resin had already thermally degraded, suggesting minimal effects of mycelium-produced char on the thermal degradation behaviour of epoxy.
- The char yield obtained from mycelium-containing epoxy composites was relatively higher than that of the neat epoxy, particularly at temperatures above 418 °C. This increase in char was attributed to the intrinsic char-forming ability of mycelium due the presence of hydroxyl-terminated polysaccharides and the superior thermal stability of deacetylated mycelium.
- Improved char formation in mycelium-containing epoxy composites paves the way for the development of polymer coating formulations for thermal protection of flammable composite substrates.
- Unmodified and deacetylated mycelium achieved the V-1 and V-0 UL94 ratings suggesting superior resistance to flame spread. Further, when mixed with epoxy resin, mycelium-containing epoxy composites had a reduced flame spread rate to neat epoxy suggesting improved fire safety ratings.

The integration of mycelium into epoxy does not alter the thermal degradation pathways of the polymer matrix. However, improved char formation in mycelium-containing epoxy composites suggests the potential for developing sacrificial surface coatings for fireproofing flammable substrates. Upon exposure to extreme temperatures, mycelium-containing polymer coatings would thermally decompose to form a residual char layer that then acts as a protective barrier for the underlying substrate. Moreover, mycelium-containing epoxy composites had reduced flame spread rates compared to the neat epoxy polymer. These findings contribute to our understanding of the interaction between bio-derived mycelium and epoxy resin, guiding the development of sustainable and environmentally friendly fire-retardant solutions. In conclusion, this study expands our knowledge of the interaction between mycelium and epoxy resin, aiding in the design and development of sustainable, non-toxic, and biodegradable fire safety solutions.

## Data Availability

The datasets used and/or analysed during the current study available from the corresponding author on reasonable request.
